# The Prognostic Immune and Nutritional Index as a Predictor of Survival in Resected Non-Small Cell Lung Cancer

**DOI:** 10.3390/medicina61101763

**Published:** 2025-09-29

**Authors:** Soomin An, Sehyun Kim, Wankyu Eo, Sookyung Lee

**Affiliations:** 1 Department of Nursing, Dongyang University, Gyeongbuk 36040, Republic of Korea; sue339@naver.com; 2 Graduate School, Dankook University, Yongin 16890, Republic of Korea; 3 Department of Internal Medicine, College of Medicine, Kyung Hee University, Seoul 05278, Republic of Korea; 4 Department of Clinical Oncology, College of Korean Medicine, Kyung Hee University, Seoul 05278, Republic of Korea

**Keywords:** non-small cell lung cancer, pulmonary surgical procedures, immunity, nutritional status

## Abstract

*Background and Objectives*: The prognostic immune and nutritional index (PINI), derived from serum albumin levels and absolute monocyte counts, has demonstrated prognostic value in gastrointestinal cancers. However, its role in non-small cell lung cancer (NSCLC) remains unclear. This study assessed the prognostic utility of the PINI for overall survival (OS) in patients with stage I–IIIA NSCLC undergoing curative-intent resection. *Methods*: This was a retrospective cohort study that included 522 patients. Cox proportional hazards models were used to evaluate the association between PINI and OS along with clinical and hematologic variables. Model performance was assessed using the concordance index (C-index), integrated area under the curve (iAUC), continuous net reclassification improvement (cNRI), integrated discrimination improvement (IDI), nomogram construction, and calibration curves. *Results*: In the multivariate analysis, the PINI remained an independent predictor of OS, along with age, American Society of Anesthesiologists physical status, stage, pleural invasion, and the modified Shine–Lal index. The full model (FM), incorporating all these variables, outperformed the baseline model (BM) that was based solely on stage (C-index: 0.841 vs. 0.692; iAUC: 0.804 vs. 0.663; both *p* < 0.001). Compared with the intermediate model (IM), which included all FM variables except the PINI, the FM demonstrated modest but statistically significant improvements (C-index: 0.841 vs. 0.820, *p* = 0.012; iAUC: 0.804 vs. 0.793, *p* = 0.001). At 3- and 5-year time points, the FM still yielded superior risk reclassification over the BM and IM, as indicated by improvements in IDI and cNRI. A nomogram based on the FM showed good calibration with the observed survival outcomes. *Conclusions*: The PINI is an independent and clinically meaningful prognostic biomarker in patients with stage I–IIIA NSCLC undergoing curative surgery. Incorporating the PINI into the BM or IM improved risk discrimination and reclassification, supporting its potential use in personalized prognostic assessment. However, external validation is warranted.

## 1. Introduction

Lung cancer remains the leading cause of cancer-related mortality worldwide, with an estimated 2.48 million new cases and 1.82 million deaths reported in 2022 [1]. Non-small cell lung cancer (NSCLC) accounts for approximately 85% of all lung cancer cases [2]. Surgical resection remains the cornerstone of curative treatment for stage I–IIIA NSCLC and offers the best chance for long-term survival. Despite advancements in surgical and perioperative care, clinical outcomes remain heterogeneous, with a substantial proportion of patients experiencing early recurrence or a poor prognosis. For instance, 5-year overall survival (OS) rates range from 60% to 74% for stage I, 47% to 55% for stage II, and approximately 38% for stage IIIA [3]. This variability underscores the need for improved prognostic tools to better stratify risks and guide personalized postoperative management.

The tumor-node-metastasis (TNM) staging system remains the cornerstone for assessing disease extent and guiding treatment decisions in NSCLC. Although OS generally decreases with advancing stage, substantial outcome variability among patients at the same stage highlights the limitations of anatomical staging in fully capturing tumor biology and host-related factors [4,5,6]. Clinical variables such as age, performance status, and smoking history add an important prognostic context, yet they often fail to reflect the underlying biological heterogeneity [7,8,9,10]. Similarly, pathological features, including histological subtype, tumor size, and pleural invasion, primarily indicate local tumor burden rather than systemic disease behavior [8,9,10,11,12,13,14,15,16]. These limitations have driven a growing interest in integrating systemic biomarkers that reflect the host’s immune, inflammatory, and nutritional status to improve risk stratification and enhance prognostic accuracy in NSCLC.

The prognostic immune and nutritional index (PINI) combines markers of nutritional and immune-inflammatory statuses by integrating serum albumin (ALB) level and absolute monocyte count (AMC). It is calculated using the formula: [ALB (g/dL) × 0.9] − [AMC (/μL) × 0.0007]. The PINI has been validated as an independent prognostic marker in gastrointestinal malignancies [17,18,19,20]. Jung et al. first reported its potential for predicting survival outcomes in colorectal cancer (CRC) [17], and subsequent studies confirmed its prognostic utility in patients with stage I–III CRC [18,19]. As regards gastric cancer, An et al. demonstrated that adding the PINI to a baseline model (BM), which is based on stage alone, or an intermediate model (IM), which includes age, body mass index (BMI), frailty index, stage, type of gastrectomy, and anemia, significantly enhanced the predictive performance, as evidenced by improvements in both the concordance index (C-index) and integrated area under the curve (iAUC), underscoring its additive value in survival prediction [20].

Despite these promising findings in colorectal and gastric cancers, the prognostic relevance of PINI in NSCLC remains unestablished. Extending its application to NSCLC is clinically important, as NSCLC differs from gastrointestinal cancers in tumor biology, host inflammatory response, and treatment paradigms. Demonstrating the utility of PINI in this distinct context addresses a key knowledge gap and highlights its potential as a broadly applicable biomarker across diverse malignancies. Notably, ALB level is a well-recognized prognostic marker for NSCLC, and it reflects nutritional status and systemic inflammation [21,22,23,24,25,26]. Similarly, elevated AMC has been associated with tumor progression through mechanisms such as immune suppression, angiogenesis, and tumor proliferation [27,28,29,30,31,32,33]. By combining ALB level and AMC, the PINI serves as a biologically integrated index that may capture both the nutritional and inflammatory landscapes of the patient, potentially enabling a more refined risk stratification in NSCLC [7,34,35,36].

This study aimed to evaluate the prognostic utility of the PINI as a continuous variable in patients with stage I–IIIA NSCLC undergoing curative-intent resection. Using real-world data and rigorous statistical methods, we investigated whether the inclusion of the PINI enhances survival prediction beyond conventional clinicopathological factors, thereby supporting its use in individualized prognostic modeling for stage I–IIIA NSCLC.

## 2. Materials and Methods

### 2.1. Study Population

This retrospective study included consecutive patients with NSCLC who underwent curative-intent surgical resection at Kyung Hee University Hospital at Gangdong between January 2010 and October 2024. Preoperative staging was performed using standard imaging modalities, including chest and abdominopelvic computed tomography (CT) and whole-body positron emission tomography (PET)-CT.

Eligible patients met the following criteria: (i) histologically confirmed primary NSCLC [37], (ii) stage I–IIIA disease according to the 8th edition of the TNM staging system [38]; and (iii) complete tumor resection with negative margins [39]. The exclusion criteria were as follows: (i) previous treatment for NSCLC, (ii) clinical or pathological evidence of stage IIIB or IV disease, (iii) history of other malignancies within the past 5 years, and (iv) active infections or autoimmune diseases requiring treatment.

Adjuvant therapy was administered to eligible patients with stage II or IIIA disease, primarily using platinum-based regimens such as cisplatin with vinorelbine, etoposide, pemetrexed, docetaxel, or gemcitabine [40]. Follow-up surveillance included chest and abdominopelvic CT scans every 3–6 months for the first 3 years, every 6 months from years 4–5, and annually thereafter. Additional imaging, including PET/CT or brain magnetic resonance imaging, was performed as clinically indicated.

This study was approved by the Institutional Review Board of Kyung Hee University Hospital at Gangdong (IRB No. 2025-07-024). The requirement for informed consent was waived owing to the retrospective design of the study.

### 2.2. Baseline Clinical Characteristics

A broad range of clinicopathological and laboratory variables was evaluated for their association with OS. Clinical parameters included age; sex; smoking status; alcohol consumption; American Society of Anesthesiologists Physical Status (ASA) classification; BMI; surgical procedure; histological subtype; tumor size; presence of pleural, vascular, lymphatic, or perineural invasion; and stage. Pleural invasion (PL) status was graded from 0 to 3 based on established histopathological criteria [41]. Alcohol consumption was defined as the intake of alcohol more than once per week, regardless of quantity [42].

Laboratory variables included routine blood chemistry and complete blood count (CBC) parameters. Biochemical variables included liver function markers such as serum total protein, ALB, total bilirubin, aspartate aminotransferase, alanine aminotransferase, and C-reactive protein (CRP) levels. The hematological indices evaluated included white blood cell (WBC) count, absolute neutrophil count (ANC), absolute lymphocyte count (ALC), AMC, red blood cell (RBC) count, hemoglobin concentration, mean corpuscular volume (MCV), mean corpuscular hemoglobin (MCH), mean corpuscular hemoglobin concentration (MCHC), the modified Shine–Lal index (mSLI) [43], platelet count, and the PINI.

All blood chemistry and CBC measurements, including serum ALB level and AMC, were obtained as part of the standard preoperative evaluation within 7 days before surgery. If multiple results were available, the value closest to the surgical date—typically collected the day before surgery—was used for analysis. Most samples were drawn in the morning under routine fasting conditions. Importantly, only pre-treatment values were analyzed; no postoperative or adjuvant therapy–related samples were included, thereby avoiding confounding effects from treatment. Blood samples for biochemical measurements were collected following standardized protocols and processed within 1 h of venipuncture. CBC samples were collected in ethylenediaminetetraacetic acid–anticoagulated tubes and similarly processed within 1 h. Hematological parameters were measured using a Beckman Coulter LH 1502 impedance analyzer (Beckman Coulter, Miami, FL, USA). Internal and external quality control procedures were routinely implemented to ensure analytical accuracy and reproducibility [44,45].

### 2.3. Statistical Analysis

The primary endpoint was OS, which was defined as the interval from curative-intent surgical resection to death from any cause or the last follow-up. To maintain granularity and generalizability, continuous variables were analyzed in their original, uncategorized form.

Univariate Cox proportional hazards models were used to screen for potential prognostic variables. Covariates with *p* values < 0.05 and satisfying the proportional hazards assumption, were considered for inclusion in the multivariate models. Multicollinearity was evaluated using variance inflation factors (VIFs). Fractional polynomial (FP) transformations were applied in both univariate and multivariate Cox regression analyses to assess the potential nonlinear associations between the PINI and OS. We selected FP over restricted cubic splines (RCS) because FP provides a flexible yet parsimonious way to model non-linear relationships, with automated selection of the best functional form. This approach reduces subjectivity in knot placement, fits the data well, and allows straightforward interpretation of hazard ratios [46].

Model discrimination was primarily evaluated using the C-index and iAUC. Model differences were tested via 1000 bootstrap resamples. To quantify the incremental prognostic value, we computed the integrated discrimination improvement (IDI) and continuous net reclassification improvement (cNRI) at 3- and 5-year time points. Decision curve analysis (DCA) was also performed at both time points to assess the net clinical benefit across a range of threshold probabilities [47].

Time-dependent C-indices were computed monthly over a 10-year horizon using bootstrapped cross-validation (1000 resamples). To minimize overfitting, the models were trained on one half of the dataset and validated on the other.

A prognostic nomogram was derived from the full model (FM) and internally validated using 1000 bootstrap replicates. Calibration curves were used to evaluate the agreement between the predicted and observed survival probabilities [47].

To identify the clinical and laboratory variables most strongly associated with the PINI, we employed least absolute shrinkage and selection operator (LASSO) regression. This technique applies an L1 penalty to enable variable selection and regularization, thus highlighting relevant predictors while reducing overfitting. The optimal penalty parameter (lambda) was selected via 10-fold cross-validation. Model performance was summarized using R-squared, root mean squared error (RMSE), and mean squared error (MSE). Features with non-zero coefficients were interpreted as contributing meaningfully to the PINI. To further enhance interpretability, SHapley Additive exPlanations (SHAP) analysis was conducted. The SHAP values provided model-agnostic estimates of each variable’s contribution to the predicted PINI. Summary plots were used to visualize the overall feature importance, whereas dependence plots illustrated the interaction effects, particularly between ALB level and AMC, the two components of the PINI.

All analyses were performed using R version 4.4.0, and statistical significance was defined as a two-sided *p* value < 0.05.

## 3. Results

### 3.1. Clinicopathological Characteristics of Study Participants

Five hundred and twenty-two patients were included in this study. The majority were of East Asian ethnicity (98.1%, *n* = 512), with a small proportion of Caucasians (1.9%, *n* = 10). Regarding disease stage, 377 patients (72.2%) were classified as stage I, 76 (14.6%) as stage II, and 69 (13.2%) as stage IIIA (Table 1).

### 3.2. Cox Regression Analysis for Predictors of Overall Survival

The median follow-up duration was 45.7 months. Univariate Cox regression was performed to screen for potential prognostic variables across demographic, clinical, pathological, biochemical, hematological, and composite domains. Candidate variables included age, sex, smoking and alcohol use, ASA, BMI, surgical approach, histological subtype, tumor characteristics (tumor size, PL, and lymphatic/vascular/perineural invasion), stage, serum chemistry (i.e., liver function tests and CRP), hematological parameters (i.e., WBC count, ANC, AMC, ALC, RBC count, hemoglobin level, MCV, MCH, MCHC, mSLI, and platelet count), and the PINI.

**Figure 1:**
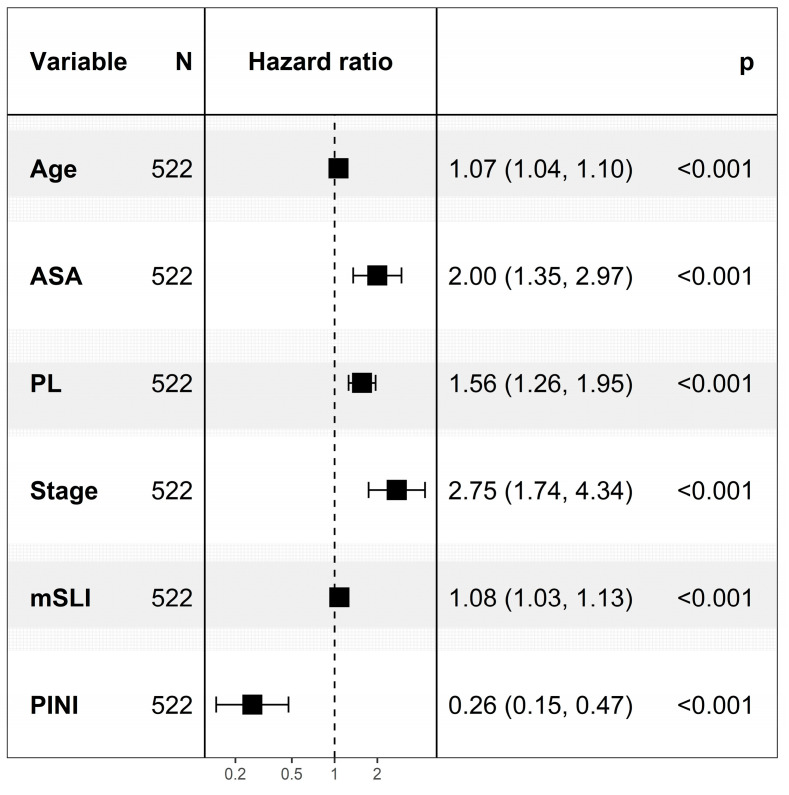
Cox regression analysis for predictors of overall survival. mSLI, modified Shine–Lal index; PINI, prognostic immune and nutritional index; PL, pleural invasion.

In multivariate modeling, age (HR, 1.07; *p* < 0.001), ASA (HR, 2.00; *p* < 0.001), PL (HR, 1.56; *p* < 0.001), stage (HR, 2.75; *p* < 0.001), mSLI (HR, 1.08; *p* < 0.001), and the PINI (HR, 0.26; *p* < 0.001) were significant with a C-index of 0.841, constituting the FM. The VIFs for the variables were as follows: age, 1.06; ASA, 1.05; stage, 1.12; PL, 1.11; mSLI, 1.05; and PINI, 1.11, indicating low multicollinearity among all covariates (Figure 1).

**Figure 2:**
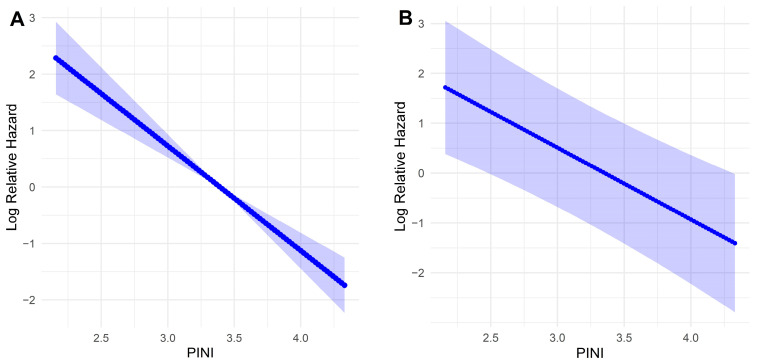
Association between the prognostic immune and nutritional index and the log-relative hazard of death using fractional polynomial Cox regression. (**A**) Univariate model; (**B**) Multivariate model adjusted for clinical covariates. Shaded areas represent 95% confidence intervals.

In the univariate model, FP analysis demonstrated a linear inverse association between the PINI and log-relative hazard, indicating that higher PINI values were associated with improved survival. This linear relationship remained consistent after multivariable adjustment for key clinical covariates, including age, ASA, PL, stage, and the mSLI. These results support the role of the PINI as a continuous and independent prognostic factor for OS in patients with NSCLC (Figure 2).

### 3.3. Nomogram Derived from the FM to Predict 3- and 5-Year Survival

**Figure 3:**
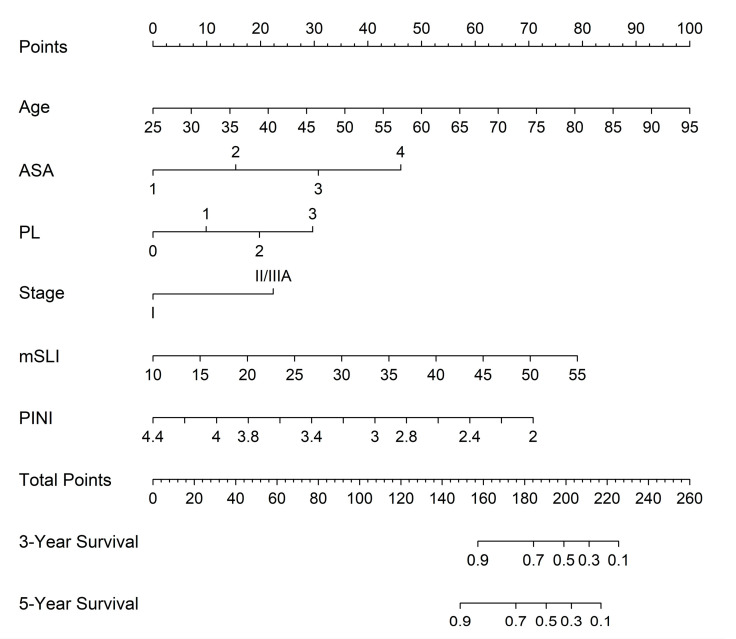
Predictive nomogram for overall survival based on the full model. mSLI, modified Shine–Lal index; PINI, prognostic immune and nutritional index; PL, pleural invasion.

A nomogram derived from the FM was constructed to generate individualized estimates of the 3- and 5-year OS rates. Incorporating key prognostic variables, such as age, ASA, PL, stage, mSLI, and PINI, is a practical and user-friendly tool to enhance personalized risk assessment and guide postoperative management (Figure 3).

**Figure 4:**
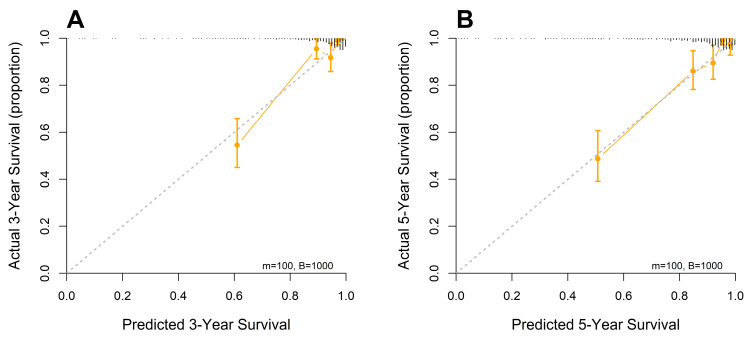
Calibration curve analysis for predicting 3-year (**A**) and 5-year (**B**) overall survival based on the full model.

Calibration plots at 3 and 5 years demonstrated close concordance between the predicted survival probabilities and actual outcomes, indicating excellent agreement and minimal deviation from the ideal 45-degree reference line. The predictive accuracy of the model was further supported by bootstrap resampling (1000 iterations), which yielded calibration curves with negligible bias and consistent performance across the risk spectrum. These results affirm the reliability and generalizability of the nomogram for individualized survival prediction in patients with resected stage I–IIIA NSCLC (Figure 4).

### 3.4. Model Comparison for Survival Prediction: FM vs. IM vs. BM

To evaluate the incremental prognostic value of the FM incorporating the PINI, we compared it with two reference models: a BM including only stage and an IM that included all the FM variables except the PINI.

The C-index and their standard errors (SEs) were 0.841 (SE = 0.022), 0.820 (SE = 0.025), and 0.692 (SE = 0.028) for the FM, IM, and BM, respectively. Similarly, the iAUC values and their SEs were 0.804 (SE = 0.019), 0.793 (SE = 0.020), and 0.663 (SE = 0.027) for the FM, IM, and BM, respectively.

The FM significantly outperformed the BM across all performance metrics. It demonstrated a higher C-index (*p* < 0.001) and superior iAUC (*p* < 0.001), indicating stronger overall discrimination. These results highlight the superior prognostic accuracy of the FM and its value in refining risk stratification beyond traditional staging. To evaluate the incremental contribution of the PINI, the FM was compared with the IM. The FM showed a modest but statistically significant improvement in the C-index (*p* = 0.012) and iAUC (*p* = 0.001).

At the 3-year time point, the FM demonstrated significantly improved discrimination compared to the BM, with an IDI of 0.252 (*p* < 0.001), indicating a 25.2% absolute increase in predictive accuracy. The cNRI was also substantial, at 0.502 (*p* < 0.001), reflecting the markedly enhanced ability of the FM to reclassify patient risk. When the FM and IM were compared, statistically significant improvements were still observed, although they were more modest in magnitude. The FM showed an IDI of 0.054 (*p* = 0.008) and a cNRI of 0.320 (*p* = 0.020), supporting the additive prognostic value of the PINI, even after accounting for other clinical variables.

At the 5-year time point, the improvements remained significant, although they were slightly attenuated. Compared to the BM, the FM achieved an IDI of 0.230 (*p* < 0.001) and a cNRI of 0.418 (*p* < 0.001). In comparison with the IM, the FM again showed statistically significant gains, with an IDI of 0.039 (*p* = 0.018) and a cNRI of 0.238 (*p* = 0.032), reaffirming the long-term prognostic contribution of the PINI in stage I–IIIA NSCLC (Table 2).

**Figure 5:**
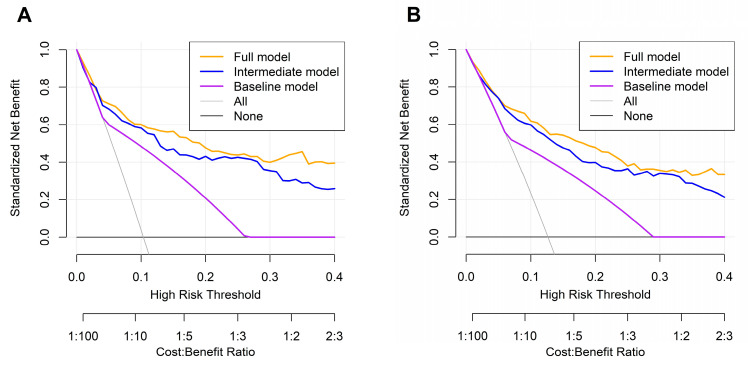
Decision curve analysis for 3-year (**A**) and 5-year (**B**) overall survival.

DCA further demonstrated that the FM consistently offered a greater net clinical benefit than both the BM and IM across a broad range of threshold probabilities for predicting both 3-year and 5-year OS. These findings suggest that incorporating additional clinical and hematological variables, particularly the PINI, enhances the ability of the model to stratify risk at the individual level. By outperforming both comparator models, the FM reduced the potential for overtreatment or undertreatment, thereby facilitating more precise and personalized postoperative management (Figure 5).

**Figure 6:**
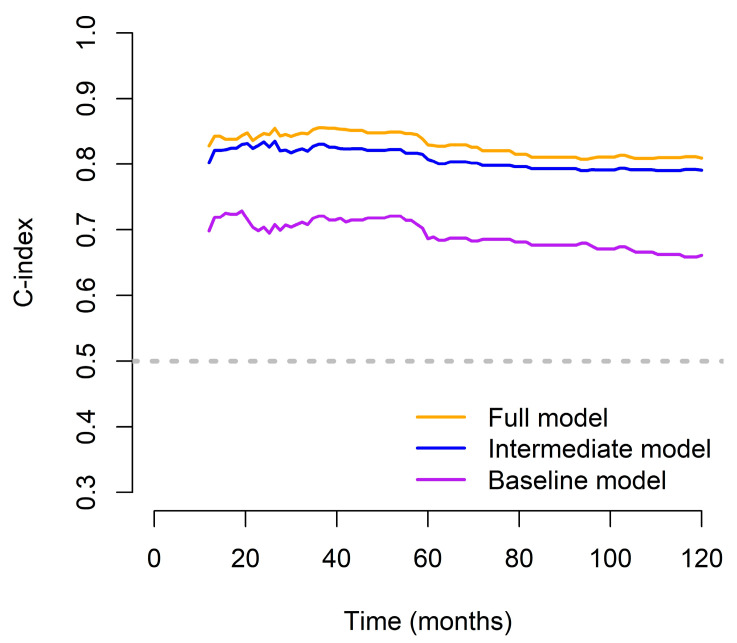
Time-dependent C-index over a 10-year period for the full, intermediate, and baseline models.

The FM demonstrated substantial and sustained improvements over both the BM and IM across the 10-year follow-up period, as reflected by consistently higher time-dependent C-index values. Notably, the BM had the lowest C-index values throughout, underscoring its limited prognostic utility when used in isolation (Figure 6).

### 3.5. PINI vs. Established Biomarkers: Model Discrimination for Survival Outcomes

To assess the prognostic utility of the PINI in comparison with ALB level and other established composite biomarkers, we constructed separate multivariate Cox models by individually adding each biomarker—ALB, neutrophil-to-lymphocyte ratio (NLR), lymphocyte-to-monocyte ratio (LMR), platelet-to-lymphocyte ratio (PLR), prognostic nutritional index (PNI), systemic immune-inflammation index (SII), systemic inflammation response index (SIRI), hemoglobin–ALB–lymphocyte–platelet (HALP) score, and CRP–ALB–lymphocyte (CALLY) index—to the IM framework, which included age, ASA, PL, stage, and mSLI [4,44,45,46,47,48,49,50,51,52,53].

**Figure 7:**
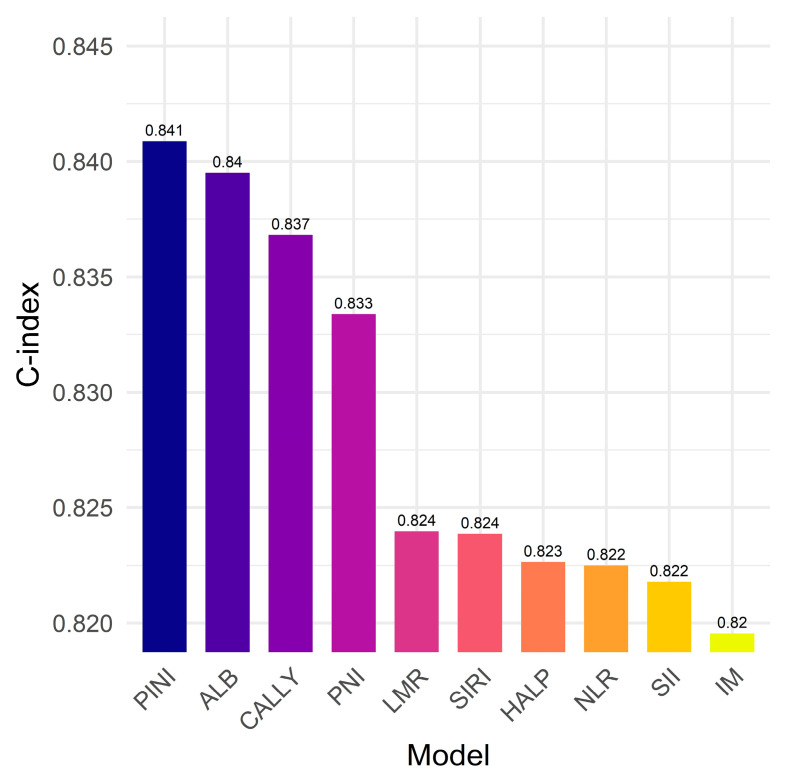
The prognostic immune and nutritional index vs. established biomarkers: Model discrimination for survival outcomes. ALB, albumin; CALLY, CRP–ALB–lymphocyte index; HALP, hemoglobin–ALB–lymphocyte–platelet; IM, intermediate model; LMR, lymphocyte-to-monocyte ratio; NLR, neutrophil-to-lymphocyte ratio; PINI, prognostic immune and nutritional index; PNI, prognostic nutritional index; SII, systemic immune-inflammation index; SIRI, systemic inflammation response index.

Among all models, the model incorporating the PINI achieved the highest C-index (0.841), indicating superior discriminative performance. Models including the ALB level (C-index = 0.840), CALLY (C-index = 0.837), and PNI (C-index = 0.833) also performed well, whereas those with the LMR, SIRI, HALP, NLR, and SII showed more modest improvements (C-index range: 0.822–0.824). These results suggest that although several inflammation- and nutrition-based biomarkers contribute to survival prediction, the PINI provided the most robust prognostic value in this cohort (Figure 7).

### 3.6. Factors Affecting the PINI

LASSO regression and SHAP analysis were performed to elucidate factors influencing the PINI. Among 30 candidate variables, only ALB level (coefficient: 0.8799) and AMC (coefficient: −0.0007) retained nonzero coefficients in the LASSO model, reinforcing their exclusive roles in defining the PINI. The predictive performance of the model was excellent, with an R-squared of 0.999, RMSE of 0.0117, and MSE of 0.00014.

**Figure 8:**
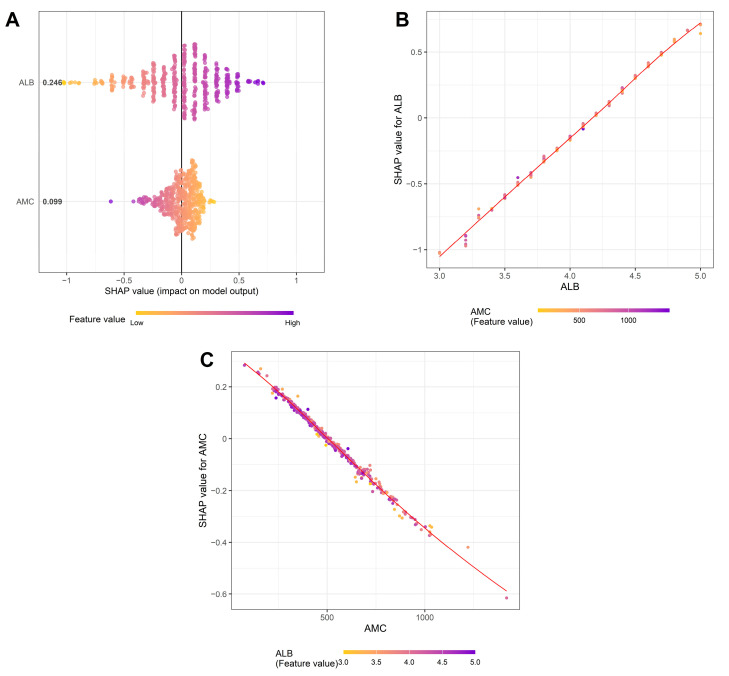
SHapley Additive exPlanations (SHAP) analysis of top features affecting the prognostic immune and nutritional index. (**A**) SHAP summary plot showing the overall impact and direction of key predictors. (**B**) Dependence plot for albumin (ALB), colored by absolute monocyte count (AMC). (**C**) Dependence plot for AMC, colored by ALB level. Red diagonal lines demonstrate the line of best fit.

SHAP analysis further revealed that ALB level contributed more strongly to the PINI than AMC (mean SHAP value: 0.246 vs. 0.099); however, both variables had substantial and opposing effects: ALB level was positively associated, while AMC was negatively associated with SHAP values of the PINI (Figure 8A). Notably, SHAP dependence plots indicated a strong interactive effect between ALB level and AMC. In the ALB dependence plot (Figure 8B), the SHAP values for ALB level (i.e., how much ALB contributed to the PINI score) increased almost linearly with increasing ALB concentration, and this effect was amplified in the context of low AMC values. Conversely, the AMC dependence plot (Figure 8C) demonstrated that the negative contribution of AMC to the PINI score became more pronounced at higher ALB levels, indicating a potential interaction between AMC and ALB level. These findings suggest that the prognostic strength of the PINI arises not only from the additive effects of its components but also from a synergistic interplay between AMC and ALB level. This interaction likely contributes to the superior prognostic utility of the PINI compared to its individual components.

## 4. Discussion

The FM, which incorporated the PINI along with key clinical and hematologic variables, demonstrated significantly better predictive performance than the BM that included only stage. This was evidenced by higher C-index and iAUC values, as well as substantial improvements in risk discrimination and reclassification, as measured by the IDI, cNRI, and DCA. When compared to the IM, which included the same clinical variables except the PINI, the FM continued to show statistically significant gains across all performance metrics. Although the incremental improvement in C-index with the addition of PINI (0.841 vs. 0.820) may appear modest, it was statistically robust and consistent across complementary measures, including iAUC, IDI, cNRI, and DCA. Importantly, even small improvements in discrimination within an already strong clinical model can be clinically meaningful, particularly near decision thresholds that guide adjuvant treatment or postoperative surveillance. These findings underscore the added prognostic value of the PINI in enhancing survival prediction among patients with stage I–IIIA NSCLC undergoing curative-intent resection.

Furthermore, the FM consistently outperformed both the BM and IM in time-dependent C-index analyses over a 10-year follow-up period. The most substantial performance gains were observed within the first 5 years and remained stable thereafter. Although the FM maintained a pronounced advantage over the BM throughout the study period, it also demonstrated consistently superior performance compared to the IM, albeit with a more modest but sustained margin. The progressive decline in the C-index of the BM over time underscores the limitations of staging as a standalone prognostic tool. Although staging remains essential for baseline risk stratification, it does not account for dynamic patient-specific factors such as systemic inflammation, nutritional decline, treatment response, or emerging comorbidities. These evolving influences likely have an increasing impact on long-term survival, thereby diminishing the predictive utility of static anatomical staging alone. Taken together, these findings highlight the value of integrative prognostic models that combine conventional staging with dynamic biomarker inputs to enable more accurate and durable risk stratification.

The prognostic impact of PINI on OS was evaluated using both univariate and multivariate Cox models, employing primarily FP to flexibly model the potential non-linear relationship. In the univariate analysis, PINI demonstrated a clear inverse association with the log relative hazard of death, with higher PINI values corresponding to lower risk. After adjusting for key clinical covariates (age, ASA, PL, stage, and mSLI), the association between PINI and survival remained consistent, supporting the independent prognostic value of PINI. The adjusted curves show that the FP method modeled the relationship as strictly linear. These findings underscore the robustness of PINI as a prognostic marker. While FP was used for the primary analysis, RCS was also explored and produced similar results. Notably, 90% of patients had PINI values between 2.77 and 3.92, which fell within the linear segment of the RCS curve. The concordance of results from both FP and RCS strengthens confidence in the robustness and linearity of the association between PINI and survival.

However, the underlying mechanisms by which the PINI predicts OS in patients with NSCLC remain unclear. In a study by An et al. involving patients with gastric cancer, LASSO regression retained only ALB level and AMC as predictors of the PINI. Their model exhibited excellent performance with an R^2^ of 0.9992 and an RMSE of 0.0112 on the test set, indicating an exceptionally strong fit. These findings support the notion that ALB level and AMC are the principal determinants of PINI expression and underscore their robustness as biologically grounded biomarkers [20]. Consistent with these findings, our LASSO regression analysis identified ALB level and AMC as the sole contributors among candidate variables. The model demonstrated near-perfect performance (R^2^ = 0.9990, RMSE = 0.0117, MSE = 0.00014), underscoring the dominant influence of these two parameters in determining the PINI.

Further interpretability was achieved through SHAP analysis. Dependence plots revealed that the SHAP values of ALB increased almost linearly with higher ALB levels, particularly when the AMC was low. Conversely, AMC exhibited the most negative impact on the PINI when ALB levels were high. These interactions suggest that the prognostic utility of the PINI is not merely due to the additive effects of its components but also stems from their dynamic interplay, which captures the interaction between systemic inflammation (i.e., AMC) and protein nutritional reserve (i.e., ALB level). This interaction explains why the PINI may outperform ALB level or AMC alone in predicting outcomes; it dynamically integrates both effects. The interaction between ALB level and AMC provides valuable insights into NSCLC progression. Biologically, ALB reflects nutritional status and systemic inflammation, while monocytes are implicated in immune suppression, angiogenesis, and tumor progression; together, they integrate key dimensions of host–tumor interaction that shape clinical outcomes. Taken together, these results provide a strong biological rationale for PINI as a robust, interpretable, and parsimonious prognostic index.

Integrating PINI into NSCLC management could improve risk stratification and support personalized care pathways. Its simplicity, affordability, and availability from routine blood tests make it practical for clinical use, potentially identifying high-risk patients who may benefit from intensified surveillance or tailored postoperative strategies, as supported by our nomogram and DCA. Future prospective and multicenter studies are needed to validate PINI’s role across diverse populations and refine its application in clinical decision-making.

In addition to the PINI, variables such as age, ASA, PL, stage, and the mSLI emerged as significant predictors of OS and were incorporated into the FM. Age is a widely recognized prognostic factor associated with worse outcomes, primarily owing to the presence of comorbidities and diminished physiological reserve [8,9,10,48]. Similarly, elevated ASA scores, which reflect a poorer preoperative health status, are linked to greater perioperative risk and reduced survival [48]. PL signifies more advanced disease and elevated recurrence risk [5,6]. Staging remains the cornerstone of NSCLC prognosis, with advanced stages reflecting lower survival rates owing to limited surgical options [7,8,9,10,16]. Although less extensively studied, RBC-derived markers are gaining recognition as prognostic indicators for NSCLC [49,50]. Among these, the mSLI, a composite metric calculated as (MCV^2^ × MCH) × 0.0001 and originally developed for the diagnosis of thalassemia, has recently emerged as a promising prognostic marker [43]. The present study reinforces the prognostic significance of RBC-derived indices, including the mSLI, in patients with stage I–IIIA NSCLC.

In comparative analyses, the PINI outperformed other well-established biomarkers, including the ALB level, NLR, LMR, PNI, SII, SIRI, HALP, and CALLY index, when each was evaluated within a consistent multivariate framework. Specifically, the model incorporating the PINI achieved the highest C-index (0.841), whereas the other models yielded slightly lower values, ranging from 0.822 to 0.840. These findings underscore the strong prognostic value of the PINI relative to other inflammation- and nutrition-based indices in patients with stage I–IIIA NSCLC. The reason for the limited incremental benefit observed with biomarkers other than PINI could be explained by the potential overlap in the captured prognostic information, highlighting the importance of selecting parsimonious and biologically relevant variables through rigorous multivariable modeling. In addition, the variability in previous findings across studies may stem from differences in patient populations, disease stages, or modeling approaches, particularly whether variables were treated as continuous or categorized. Overall, our results support integrating the PINI into clinical prognostic models and emphasize the need for careful biomarker selection to enhance predictive accuracy without redundancy.

The principal strength of this study lies in its focused evaluation of the PINI as a prognostic biomarker in patients with stage I–IIIA NSCLC undergoing curative-intent surgical resection. To our knowledge, this is the first study to demonstrate the prognostic significance of PINI in NSCLC, thereby extending its clinical relevance beyond gastrointestinal cancers. This represents more than a replication; validating PINI in NSCLC—a disease with distinct biological and clinical features—fills an important gap in biomarker research and supports its role as an accessible and versatile tool for survival prediction across cancer types. Unlike most prior biomarker studies in NSCLC, which have concentrated on advanced disease, our study uniquely investigates PINI in surgically treated stage I–IIIA patients, a population in which reliable prognostic tools are essential to guide adjuvant therapy and long-term surveillance. Biomarkers validated in advanced disease may not generalize to early-stage, surgically managed NSCLC, underscoring the clinical importance of our findings. Furthermore, we directly compared PINI with other established biomarkers. Among all models, the one incorporating PINI achieved the highest C-index (0.841), indicating superior discriminative performance. The PNI—a widely studied nutritional marker in NSCLC, calculated as 10 × ALB (g/dL) + 0.005 × ALC (/mm^3^) [51,52,53]—also showed prognostic value but was outperformed by PINI in our cohort (C-index: 0.841 vs. 0.833). This finding underscores the additive value of PINI over PNI and highlights its potential as a more comprehensive marker that integrates both nutritional and inflammatory status.

Based on the results of this study, incorporating the PINI into multivariable prognostic models may enhance both the predictive performance and risk stratification capacity. The FM demonstrated superior discrimination across various statistical metrics and provided more clinically actionable predictions through DCA and an individualized nomogram. These results suggest that the FM can help identify high-risk patients who may benefit from adjuvant therapies, closer surveillance, or tailored follow-up regimens, while simultaneously sparing low-risk individuals from overtreatment. Given its affordability, objectivity, and universal availability in routine CBC, the PINI is uniquely positioned as a pragmatic biomarker in real-world clinical settings. It avoids the limitations of expensive or technically complex assays and is not influenced by interobserver variability, offering immediate clinical applicability without increased healthcare costs. These findings underscore the potential of the PINI to complement existing staging systems and support individualized care pathways in patients with stage I–IIIA NSCLC. Although the precise biological underpinnings of the PINI remain to be fully elucidated, its robust and independent prognostic value, coupled with its accessibility, makes it a compelling candidate for broader inclusion in clinical decision-making tools and future prospective trials.

Nonetheless, this study had several limitations. First, as this was a retrospective, single-center study, the analysis was inherently subject to selection bias and residual confounding, which may limit causal inferences and generalizability. The ethnic homogeneity of the cohort (98.1% East Asian patients) further limits applicability to broader populations, given potential racial and genetic differences in tumor biology and hematologic parameters. To address these concerns, prospective multicenter studies including ethnically diverse patients and external validation cohorts are needed to confirm the prognostic utility of PINI across different settings. Despite these limitations, our findings were supported by rigorous internal validation, underscoring the robustness of PINI within the current cohort.

Another consideration is the stage distribution, with 72.2% of patients presenting with stage I disease. This predominance of early-stage cases may have contributed to the overall favorable survival outcomes observed. While this distribution reflects contemporary surgical practice, it also raises the possibility of stage-related survival bias. However, all prognostic analyses were adjusted for TNM stage, and stage was consistently retained as an independent predictor in the multivariate models. Importantly, PINI remained a significant prognostic factor even after adjusting for stage, suggesting that its utility extends beyond anatomical disease extent by capturing complementary biological dimensions influencing survival across stage subgroups.

Third, the use of baseline cross-sectional laboratory data precluded the evaluation of longitudinal changes in the PINI and their relationship with disease progression or treatment response. Postoperative PINI assessment was also challenging because adjuvant therapies, often involving chemotherapy and/or radiation, can significantly alter ALB level and AMC, leading to unstable and difficult-to-interpret values. Moreover, PINI components can be affected by non-cancer-related conditions such as infection, systemic inflammation, or autoimmune disease. In this study, we attempted to minimize such confounding by excluding patients with active infections or autoimmune disorders and by using laboratory data obtained within seven days before surgery in clinically stable patients. Nonetheless, subclinical or undetected conditions cannot be fully excluded.

Finally, although internal validation via 1000 bootstrap iterations supported the robustness of the model, the absence of external validation in independent cohorts limits its broader clinical applicability. These limitations underscore the need for future prospective multicenter studies incorporating ethnically diverse populations, serial biomarker measurements, and external validation to fully establish the prognostic utility of the PINI in NSCLC [54,55].

## 5. Conclusions

This study established the PINI as an independent and clinically meaningful biomarker for predicting OS in patients with stage I–IIIA NSCLC undergoing curative-intent surgical resection. Incorporating the PINI into the FM significantly enhanced its prognostic performance compared with that of the BM and IM, thereby improving risk stratification and supporting its potential role in guiding personalized postoperative management. Owing to its simplicity, low cost, and accessibility through routine preoperative blood tests, the PINI is a practical tool for clinical application. However, external validation in independent multi-ethnic cohorts is essential to confirm the robustness and generalizability of these findings.

## Figures and Tables

**Table 1:** Clinicopathological characteristics of study participants.

Variables		N (%) or Median (IQR)	Variables		N (%) or Median (IQR)
Age, years		69.0 (62.0–74.0)	Vascular invasion		
Sex				Yes	28 (5.4%)
	Men	303 (58.0%)		No	494 (94.6%)
	Women	219 (42.0%)	Perineural invasion		
Smoking				Yes	8 (1.5%)
	Current/Past	210 (40.2%)		No	514 (98.5%)
	Never	312 (59.8%)	TNM stage		
Alcohol consumption				IA/IB	377 (72.2%)
	Yes	132 (25.3%)		IIA/IIB/IIIA	145 (27.8%)
	No	390 (74.7%)	Protein, g/dL		7.1 (6.8–7.5)
ASA-PS			Albumin, g/dL		4.2 (4.0–4.4)
	1/2	428 (82.0%)	Total bilirubin, mg/dL		0.5 (0.4–0.6)
	3/4	94 (18.0%)	AST, U/L		22.0 (19.0–27.0)
BMI, kg/m^2^		23.9 (21.9–26.2)	ALT, U/L		17.0 (12.0–23.0)
Resection			CRP, mg/dL		0.1 (0.1–0.3)
	Sublobar resection	194 (37.2%)	WBC, per μL		6315.0 (5330.0–7460.0)
	Lobectomy	315 (60.3%)	ANC, per μL		3622.0 (2920.0–4634.0)
	Bilobectomy	6 (1.1%)	AMC, per μL		479.0 (382.0–610.0)
	Pneumonectomy	7 (1.3%)	ALC, per μL		1831.5 (1532.0–2240.0)
Histology			RBC, ×10^6^ per μL		4.3 (3.9–4.6)
	Squamous	115 (22.0%)	Hemoglobin, g/dL		13.3 (12.1–14.2)
	Non-squamous	407 (78.0%)	MCV, fL		91.8 (89.2–94.8)
Tumor size, cm		2.5 (1.7–3.5)	MCH, g/dL		30.9 (30.0–32.1)
Pleural invasion (PL)			MCHC, g/dL		33.7 (33.1–34.3)
	0	417 (79.9%)	mSLI		26.0 (24.1–28.8)
	≥1	105 (20.1%)	Platelet, ×10^3^ per μL		236.0 (202.0–278.0)
Lymphatic invasion			PINI		3.4 (3.2–3.6)
	Yes	64 (12.3%)			
	No	458 (87.7%)			

mSLI, modified Shine–Lal index; PINI, prognostic immune and nutritional index.

**Table 2:** Model comparison for survival prediction.

Metrics	Improvement (FM vs. BM)	*p* Value (FM vs. BM)	Improvement (FM vs. IM)	*p* Value (FM vs. IM)
C-index	0.153 (0.023)	<0.001	0.022 (0.012)	0.012
iAUC	0.141 (0.009)	<0.001	0.013 (0.004)	0.001
IDI at 3 years	0.252 (0.044)	<0.001	0.054 (0.024)	0.008
cNRI at 3 years	0.502 (0.069)	<0.001	0.320 (0.088)	0.020
IDI at 5 years	0.230 (0.042)	<0.001	0.039 (0.021)	0.018
cNRI at 5 years	0.418 (0.068)	<0.001	0.238 (0.086)	0.032

Values in parentheses indicate standard errors. FM, full model; IM, intermediate model; BM, baseline model; IDI, integrated discrimination improvement; cNRI, continuous net reclassification improvement; iAUC, integrated area under the curve.

## Data Availability

The datasets presented in this study are available upon request from the corresponding author owing to ethical reasons.

## Abbreviations

The following abbreviations are used in this manuscript:

ALB	Albumin
ALC	Absolute lymphocyte count
ALT	Alanine aminotransferase
AMC	Absolute monocyte count
ANC	Absolute neutrophil count
ASA	American society of anesthesiologists physical status
AST	Aspartate aminotransferase
AUC	Area under the curve
BMI	Body mass index
CALLY	CRP-albumin-lymphocyte
C-index	Concordance index
cNRI	Continuous net reclassification improvement
CRP	C-reactive protein
DCA	Decision curve analysis
DFS	Disease-free survival
FP	Fractional polynomial
HALP	Hemoglobin-albumin-lymphocyte-platelet
HR	Hazard ratio
iAUC	Integrated AUC
IDI	Integrated discrimination improvement
IQR	Interquartile range
LASSO	Least absolute shrinkage and selection operator
LMR	Monocyte-to-lymphocyte ratio
MCH	Mean corpuscular hemoglobin
MCHC	Mean corpuscular hemoglobin concentration
MCV	Mean corpuscular volume
mSLI	Modified Shine–Lal index
NLR	Neutrophil-to-lymphocyte ratio
NSCLC	Non-small cell lung cancer
OS	Overall survival
PINI	Prognostic immune and nutritional index
PL	Pleural invasion
PLR	Platelet-to-lymphocyte ratio
PNI	Prognostic nutritional index
RBC	Red blood cell
SHAP	SHapley Additive exPlanations
